# Association of Geriatric Emergency Department Care With Patient Outcomes in Older Adults

**DOI:** 10.1093/geroni/igaf122.4391

**Published:** 2025-12-31

**Authors:** Yuting Qian, Cameron Gettel, Jasmine Su, Elyssa Grogan, Inessa Cohen, Craig Rothenberg, Xi Chen, Ula Hwang

**Affiliations:** Yale School of Public Health, New Haven, Connecticut, United States; Yale School of Medicine, New Haven, Connecticut, United States; New York University School of Medicine, New York, New York, United States; NYU Langone Health, New York, New York, United States; Yale University, Yale University, Connecticut, United States; Yale University School of Medicine, New Haven, Connecticut, United States; Yale University, New Haven, Connecticut, United States; NYU Grossman School of Medicine, New York, New York, United States

## Abstract

Since 2018, the Geriatric Emergency Department (GED) accreditation program has recognized EDs that provide high-quality care tailored to older adults. Despite its considerable expansion in recent years, no studies have evaluated the impact of GED care on patient-centered outcomes using national data. Our objective was to determine whether GED care is associated with improved outcomes among older adults. We used the 2018-2021 Health and Retirement Study-Medicare linked data of adults aged≥65 years, weighted for national estimates. Receipt of GED care was defined as having an ED visit at a GED (versus not). Patient-level analyses were conducted using each individual’s most recent ED visit. Multivariable logistic regression models were used to estimate associations between receipt of GED care and outcomes of hospital admission and 30-day mortality, adjusting for patient demographics, socioeconomic status, health conditions, and hospital-level characteristics. Among 4,570 older adults – representing 25,317,444 adults nationally – with an ED visit, 270 (5.9%) received GED care. Compared with those treated in non-GEDs, patients treated in GEDs had significantly lower odds of hospital admission (OR, 0.710; 95% CI, 0.505-0.998), primarily among adults aged 65-80 years (OR, 0.565; 95% CI, 0.336-0.952) and non-Hispanic White individuals (OR, 0.614; 95% CI, 0.410-0.920). Among non-Hispanic White individuals, GED care was also associated with a significant reduction in 30-day mortality (OR, 0.554; 95% CI, 0.324-0.945). This is the first study using national data to demonstrate that GED care significantly improves outcomes for older adult subgroups. Broader implementation can enhance reach and impact across programs and diverse populations.

